# Development and validation of a deep learning radiomics model based on ultrasound and clinical features to predict prognosis in elderly patients with advanced pancreatic cancer after HIFU therapy

**DOI:** 10.1186/s12876-026-04716-6

**Published:** 2026-03-05

**Authors:** Yumei Liu, Yongshuo Ji, Junqiu Zhu, Linglin Zhu, Yanfei Zhu, Hong Zhao, Zhijun Bao

**Affiliations:** 1 High-Intensity Focused Ultrasound Center of Oncology Department, Huadong Hospital Affiliated to Fudan University, 139 West Yan’an Road, Jing’an, Shanghai, 200000 China; 2 Department of Gerontology, Huadong Hospital Affiliated to Fudan University, 221 West Yan’an Road, Jing’an, Shanghai, 200040 China; 3 Shanghai Key Laboratory of Clinical Geriatric Medicine, Shanghai, China; 4 Shanghai Institute of Geriatrics and Gerontology, Shanghai, China

**Keywords:** Pancreatic cancer, HIFU, Prognosis, Deep learning, Radiomics, Ultrasonography, Aged

## Abstract

**Background:**

This study aimed to construct an artificial intelligence model based on ultrasound radiomics and deep learning, integrating clinical features to develop a fusion model for individualized prediction of survival outcomes in elderly patients with advanced pancreatic cancer receiving high-intensity focused ultrasound (HIFU) treatment.

**Methods:**

This retrospective study enrolled elderly patients with advanced pancreatic cancer admitted to Huadong Hospital Affiliated to Fudan University from March 2015 to March 2024, randomly divided into training and validation cohorts in a 7:3 ratio. Patients were categorized into four groups based on treatment modality: HIFU alone, HIFU combined with ^125^I seed implantation, HIFU combined with chemotherapy, and triple therapy (HIFU + ^125^I + chemotherapy). Traditional radiomics features and deep learning features based on the ResNet architecture were extracted from pre-treatment ultrasound images. After rigorous feature selection, clinical, radiomics, deep learning, and multi-modal fusion Cox proportional hazards models were constructed. Predictive performance for overall survival and clinical utility were evaluated comprehensively.

**Results:**

This study included 250 elderly pancreatic cancer patients. Multivariate analysis identified liver metastasis, TNM stage, body weight, number of HIFU sessions, and treatment regimen as independent prognostic factors (all *p* < 0.05). Predictive models were constructed using selected clinical features, traditional radiomics and deep learning features from ultrasound images. The deep learning radiomics model demonstrated the highest C-indices in both the training and validation sets (0.735 and 0.716, respectively), outperforming the clinical model (0.664, 0.633) and the traditional radiomics model (0.658, 0.592). The combined model, integrating clinical and deep learning features, achieved the best predictive performance, with C-indices of 0.748 (training) and 0.739 (validation). Time-dependent ROC analysis further confirmed that the combined model maintained the highest AUC values for 1-year and 1.5-year survival prediction in the validation set (0.819 and 0.884, respectively), significantly enhancing the accuracy and generalizability of survival stratification.

**Conclusions:**

The ultrasound-based deep learning radiomics model demonstrated favorable performance in predicting the prognosis of elderly pancreatic cancer patients undergoing HIFU treatment, with performance superior to traditional clinical and radiomics indicators. It can facilitate more accurate individualized survival risk stratification, providing a potentially useful tool for precise treatment decision-making in advanced pancreatic cancer.

**Supplementary Information:**

The online version contains supplementary material available at 10.1186/s12876-026-04716-6.

## Introduction

Pancreatic cancer carries an extremely poor prognosis. Although the 5-year survival rate has increased from 3.1% to 10% in recent years under multidisciplinary integrated treatment models, over 80% of patients still die within months of diagnosis [1–3]. Data from the United States in 2024 indicate that 75% of patients are diagnosed at an advanced stage, with a mere 3% 5-year survival rate for this group [4]. Pancreatic cancer predominantly affects the elderly, with approximately two-thirds of patients aged 65 years or older [1, 2]. However, through enhanced cancer prevention and control measures, this trend may be mitigated or even reversed [5].

High-intensity focused ultrasound (HIFU) is a non-invasive technology that uses heat to ablate tumors. Its principle involves focusing ultrasound waves on the target tumor inside the body, generating high energy at the focal point to instantaneously reach temperatures above 60 °C, thereby inducing coagulative necrosis and apoptosis via thermal, cavitation, mechanical, and potential immune effects, without damaging surrounding organs [6, 7]. HIFU has been used to treat advanced pancreatic cancer, demonstrating good safety and tolerability, making it particularly suitable for elderly and frail patients [8, 9]. HIFU is generally not intended as a curative treatment modality, but rather as a non-invasive, locally palliative intervention aimed at achieving local tumor control, pain relief, and serving as a complement to systemic therapy. In selected patients, it may improve quality of life and prolong survival. Beyond achieving local tumor control and alleviating tumor-related pain, HIFU has also been shown to improve progression-free survival and overall survival [10, 11]. Studies conducted in Asia report overall survival times of 6 to 11 months and median progression-free survival of 5 to 8.4 months for pancreatic cancer patients treated with HIFU [12–14].

## HIFU combined with systemic chemotherapy

Systemic chemotherapy remains the cornerstone treatment for advanced pancreatic cancer, but its efficacy is limited. First-line regimens such as nab-paclitaxel plus gemcitabine or mFOLFIRINOX yield 1-year survival rates of only 18%–20% [15–17]. Recent studies indicate that combining HIFU with chemotherapy can significantly enhance efficacy. A meta-analysis demonstrated that combination therapy improved overall survival (HR = 0.40) and increased the 1-year survival rate (OR = 0.35) [18]. Another study reported a median survival of 13.82 months in the combination therapy group [19]. In addition, patients receiving HIFU combined with chemotherapy experienced significant pain reduction (*p* < 0.05) [20]. However, some patients still respond poorly, with response rates ranging from 33.3% to 76.6% [13, 21], highlighting the need to accurately identify potential beneficiaries prior to treatment to avoid unnecessary chemotherapy-related toxicity and economic burden, and to pursue alternative effective therapies.

### HIFU combined with radioactive iodine-125 seed implantation

Radioactive iodine-125 (¹²⁵I) seed implantation is a minimally invasive and repeatable form of local radiotherapy that provides continuous irradiation to the tumor, enhancing the radiobiological effect [22, 23]. In patients with unresectable pancreatic cancer, the clinical benefit rate of ¹²⁵I seed implantation can reach 92.3%, significantly higher than 41.7% in non-recipients (*p* < 0.01) [24]. For stage III or higher pancreatic cancer, ¹²⁵I seed implantation extends median survival to 12.8 months, with an overall response rate of 91%, outperforming conventional treatments [25]. Both ¹²⁵I seed implantation and HIFU significantly alleviate cancer pain and may serve as alternatives or supplements to opioids or nerve blocks [26, 27]. Our team’s previous research showed that patients with advanced pancreatic cancer receiving repeated HIFU combined with ¹²⁵I seed implantation achieved a median overall survival of 13.1 months, with 3-, 6-, 9-, and 12-month survival rates of 100.0%, 86.5%, 61.5%, and 53.8%, respectively. Post-treatment, patients demonstrated significantly improved KPS scores and reduced pain intensity, without serious complications [28]. Although reports on this combination strategy remain limited, it holds promise as a new treatment option for elderly patients with advanced disease. However, as ¹²⁵I seed implantation is an invasive procedure, establishing effective prognostic prediction models to accurately select potential beneficiaries prior to treatment is essential.

### Artificial intelligence models for prognosis of HIFU treatment in elderly pancreatic cancer patients

Current prognostic assessment for pancreatic cancer mainly relies on TNM staging, but its clinical utility is limited: survival varies widely within the same stage, and N staging requires postoperative pathology, which is not applicable to non-surgical patients [29, 30]. Other indicators such as CA19-9 and radiomics lack standardized consensus [31, 32]. A CT-based radiomics study found that lower skeletal muscle index or higher ECOG scores were associated with significantly shorter survival after HIFU, offering a new prognostic perspective for elderly patients [33]. Radiomics and deep learning can capture tumor heterogeneity through high-dimensional image features [34, 35], and have been applied to predict survival, therapy response, and resectability [36–38]. Ultrasound is safe, convenient, and radiation-free, making it particularly suitable for elderly patients. Several studies have combined ultrasound radiomics with clinical features to predict lymph node metastasis and treatment response in pancreatic cancer [39–41]. However, research integrating ultrasound imaging with deep learning to predict prognosis specifically in elderly pancreatic cancer patients undergoing HIFU remains scarce. Given the suitability and cost-effectiveness of ultrasound in this population, such studies are of substantial clinical relevance.

In summary, evidence for pancreatic cancer treatment remains limited, particularly for predicting HIFU efficacy in elderly patients. Existing radiomics lack unified standards. Given the low response to chemotherapy and the invasive nature of ¹²⁵I seed implantation, accurately identifying potential HIFU responders before treatment is essential. A prognostic model based on ultrasound radiomics and deep learning can provide non-invasive, individualized decision support for elderly patients. This study aims to optimize HIFU strategies and improve outcomes by establishing such a tool.

## Methods

### Study population

This retrospective study enrolled elderly patients (≥ 65 years) with advanced pancreatic cancer treated at Huadong Hospital, Fudan University, from March 2015 to March 2024. Patients were randomly divided into training and validation cohorts (7:3). Last follow-up: August 31, 2025. Inclusion criteria: (1) Age ≥ 65; (2) Stage III/IV pancreatic cancer; (3) Pre-treatment ultrasound at our hospital with visible tumor; (4) Ineligible for radical resection; (5) ≥ 1 HIFU session; (6) Treatment groups: HIFU alone, HIFU+chemotherapy, HIFU+¹²⁵I seed, or HIFU+chemotherapy+seed. No concurrent antitumor therapies. Exclusion criteria: (1) Incomplete data; (2) Poor ultrasound quality; (3) Other primary malignancies. Collected data: demographics, laboratory tests, tumor markers, pain/NRS scores, KPS, and imaging. HIFU interval: ≥4 weeks. HIFU+chemotherapy interval: ≤30 days. For triple therapy, chemotherapy–seed interval: 1–8 weeks, with ≥ 2 chemotherapy cycles. HIFU was performed before seed implantation due to pain control needs and minimally invasive priority. The clinical data collection form (Supplementary File 1) was designed for this study. Approval: Ethics Committee of Huadong Hospital, Fudan University (20250027, Shanghai). Informed consent waived due to retrospective design.

### Artificial intelligence related analysis

**Fig. 1:**
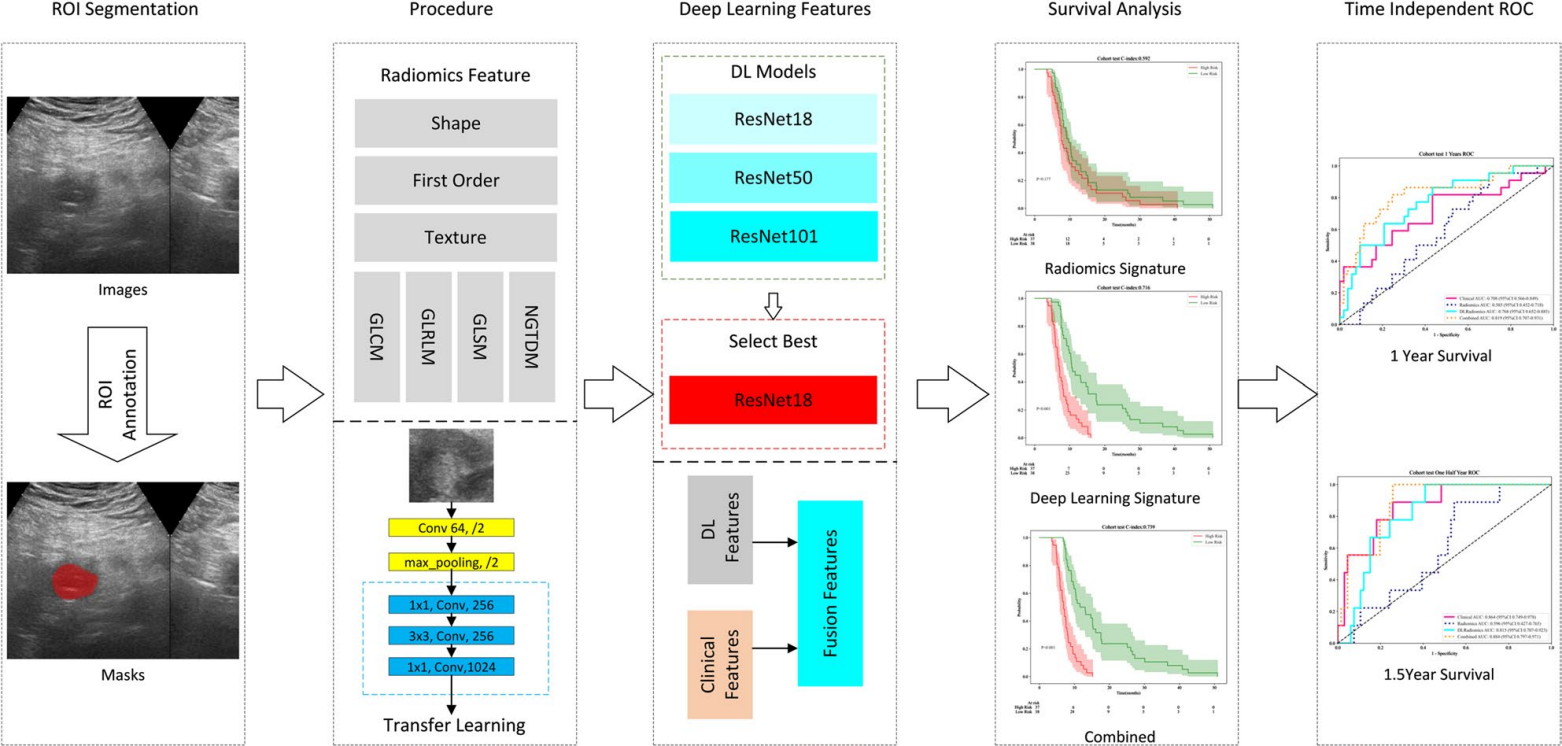
Workflow of this study

This study combined traditional radiomics and deep learning to extract features from the largest region of interest (ROI). Radiomics features were from manual ROIs; deep learning features from the largest ROI identified by the neural network. After feature selection, redundant features were removed and prognostic variables retained. A Cox model was built for survival prediction. Patients were stratified into high- and low-risk groups by median risk score; survival differences were tested using log-rank. Time-dependent ROC curves at 1 and 1.5 years assessed model discrimination. Figure 1 shows the workflow.

### Acquisition of ultrasound images

Ultrasound examination of the pancreas was performed within 2 weeks before treatment using a MyLab 70 system (Esaote, Italy) with a 3–8 MHz convex probe. Lesion images were recorded in two orthogonal planes in DICOM format.

### ROI delineation

Regions of interest (ROIs) were manually delineated on the largest cross-sectional ultrasound image using ITK-SNAP. Two abdominal radiologists with > 5 years of experience performed blind annotations. Inter-observer consistency was assessed using the Dice coefficient (threshold ≥ 0.85). Discrepancies were resolved by a third expert (> 20 years of experience).

### Clinical feature selection

Baseline data included ultrasound characteristics, clinical variables, and blood indicators. Univariate and multivariate Cox regression (*p* < 0.05) identified independent prognostic clinical features for model construction.

### Radiomics analysis pipeline

#### Radiomics feature extraction

Radiomics features were extracted from ROIs using PyRadiomics (v3.0.1) following IBSI guidelines. Features included shape, first-order statistics, and texture matrices (GLCM, GLRLM, GLSZM, NGTDM). Images underwent resolution and intensity normalization. Wavelet and Laplacian of Gaussian (LoG) filters were applied to enhance feature expression.

#### Feature selection

A multistage selection process was used: (1) Pearson correlation eliminated redundant pairs (|r| > 0.9); (2) univariate Cox regression (*p* < 0.05) screened survival-associated features; (3) LASSO-Cox with 10-fold cross-validation selected the final prognostic feature subset.

### Deep learning analysis pipeline

#### Data preprocessing

The slice with the largest ROI was cropped to its minimum bounding rectangle and normalized using Z-score. Online augmentation (random cropping, horizontal/vertical flipping) was applied only to the training set.

### Model training

A CNN-based classification model was built using transfer learning (ImageNet pretrained weights). ResNet18, ResNet50, and ResNet101 were evaluated; the optimal model was selected based on validation performance. Optimization: SGD with softmax cross-entropy loss and cosine annealing learning rate scheduling.

### Feature extraction and fusion

Deep features were extracted from the global average pooling layer of the trained CNN. Traditional radiomics features were extracted from the same ROI using PyRadiomics. The two feature sets were fused via feature space alignment; collinear variables were removed. The final feature subset was input into a Cox proportional hazards model.

### Model construction

Cox models were built stepwise: Radiomics model: based on selected radiomics features. Deep learning model: based on CNN-derived deep features. Clinical model: based on significant clinical variables (*p* < 0.05) from multivariate Cox regression. Combined model: integrating significant clinical predictors with radiomics/deep learning outputs.

### Nomogram establishment

The best-performing model was visualized as a nomogram. Each variable was assigned points based on regression coefficients; total points predicted 1-, 2-, and 3-year overall survival.

### Evaluation metrics

Survival analysis used L2-regularized Cox regression. Patients were stratified by median hazard ratio; survival differences were assessed via Kaplan-Meier and log-rank tests. Model performance was evaluated using AUC (95% CI), accuracy, sensitivity, and specificity. Decision curve analysis (DCA) quantified clinical net benefit across threshold probabilities.

### Statistical analysis

Continuous variables were tested for normality (Shapiro–Wilk) and compared using t-test or Mann–Whitney U test. Categorical variables were compared using the chi-square test. No significant differences were found between training and validation sets (*p* > 0.05), indicating balanced partitioning. Univariate and multivariate Cox regression were used for prognostic factor analysis. Statistical analyses were performed using Statsmodels (v0.13.2). Radiomics features were extracted via PyRadiomics (v3.0.1). Machine learning models were built with Scikit-learn (v1.0.2); deep learning models with PyTorch (v1.11.0) and CUDA/cuDNN acceleration. All tests were two-sided, with significance set at α = 0.05.

## Results

### Patient baseline characteristics

A total of 250 patients were randomly divided into training (*n* = 175) and test (*n* = 75) sets (7:3). No significant differences in baseline variables were found between the two groups (*p* > 0.05) (Table 1). The cohort included 154 males and 96 females, aged 65–91 years (mean 70.7 ± 6.1 years). As of August 31, 2025, median follow-up was 8.2 months (95% CI: 7.664–8.736); survival ranged from 3.5 to 50.9 months.

**Table 1:** Baseline characteristic of our cohort

Feature_name	ALL	Train	Test	*p*-Value
Age(years)	70.66 ± 6.11	71.10 ± 6.31	69.65 ± 5.53	0.068
Diameter(mm)	45.34 ± 15.61	45.81 ± 16.45	44.23 ± 13.51	0.725
Size(mm^2^)	1652.40 ± 1202.95	1692.33 ± 1309.12	1559.25 ± 909.36	0.982
NRS	2.63 ± 2.51	2.58 ± 2.56	2.75 ± 2.41	0.535
NRS one month after treatment	1.86 ± 2.08	1.88 ± 2.07	1.80 ± 2.11	0.708
CA19-9(U/ml)	2172.72 ± 3129.45	2076.05 ± 2962.77	2398.29 ± 3498.54	0.429
CA125(U/ml)	308.32 ± 767.43	295.11 ± 778.84	339.16 ± 744.34	0.505
Weight(kg)	59.84 ± 7.96	59.29 ± 7.93	61.13 ± 7.93	0.099
Height(m)	1.69 ± 0.08	1.68 ± 0.08	1.70 ± 0.08	0.138
BMI	21.02 ± 2.27	20.95 ± 2.27	21.18 ± 2.27	0.471
HIFU cycle count	2.36 ± 1.94	2.39 ± 1.94	2.29 ± 1.97	0.493
Gender				0.514
Male	154(61.60)	105(60.00)	49(65.33)	
Female	96(38.40)	70(40.00)	26(34.67)	
Location				0.326
Head	104(41.60)	68(38.86)	36(48.00)	
Neck	30(12.00)	21(12.00)	9(12.00)	
Body	74(29.60)	52(29.71)	22(29.33)	
Tail	42(16.80)	34(19.43)	8(10.67)	
Echo				0.731
Hypoechoic	222(88.80)	154(88.00)	68(90.67)	
Isoechoic	6(2.40)	4(2.29)	2(2.67)	
Heterogeneous echogenicity	22(8.80)	17(9.71)	5(6.67)	
Texture				1.0
Homogeneous	32(12.80)	22(12.57)	10(13.33)	
Heterogeneous	218(87.20)	153(87.43)	65(86.67)	
Boundary				0.606
Clear	91(36.40)	66(37.71)	25(33.33)	
Not clear	159(63.60)	109(62.29)	50(66.67)	
Shape				0.951
Regular	69(27.60)	49(28.00)	20(26.67)	
Irregular	181(72.40)	126(72.00)	55(73.33)	
Liver metastasis				0.824
Present	111(44.40)	79(45.14)	32(42.67)	
Absent	139(55.60)	96(54.86)	43(57.33)	
Lymph node metastasis				0.355
Present	104(41.60)	69(39.43)	35(46.67)	
Absent	146(58.40)	106(60.57)	40(53.33)	
Vascular invasion				0.725
Present	62(24.80)	45(25.71)	17(22.67)	
Absent	188(75.20)	130(74.29)	58(77.33)	
TNM				0.487
III	87(34.80)	58(33.14)	29(38.67)	
IV	163(65.20)	117(66.86)	46(61.33)	
KPS				0.29
50	3(1.20)	2(1.14)	1(1.33)	
60	10(4.00)	6(3.43)	4(5.33)	
70	38(15.20)	26(14.86)	12(16.00)	
80	122(48.80)	90(51.43)	32(42.67)	
90	75(30.00)	51(29.14)	24(32.00)	
100	2(0.80)	null	2(2.67)	
Treatment sequence				0.945
HIFU first	199(79.60)	140(80.00)	59(78.67)	
HIFU later	51(20.40)	35(20.00)	16(21.33)	
Jaundice				0.863
Present	50(20.00)	36(20.57)	14(18.67)	
Absent	200(80.00)	139(79.43)	61(81.33)	
Treatment regimen				0.301
HIFU	83(33.20)	63(36.00)	20(26.67)	
HIFU+ chemotherapy	71(28.40)	44(25.14)	27(36.00)	
HIFU+ iodine-125 seeds implantation	67(26.80)	48(27.43)	19(25.33)	
HIFU+ chemotherapy + iodine-125 seeds implantation	29(11.60)	20(11.43)	9(12.00)	
Chemotherapy regimen				0.119
No chemotherapy	150(60.00)	111(63.43)	39(52.00)	
Others (tegionine/capecitabine/cisplatin/raltitrexel, etc.)	21(8.40)	11(6.29)	10(13.33)	
mFOLFIRINOX class	28(11.20)	21(12.00)	7(9.33)	
AG class	51(20.40)	32(18.29)	19(25.33)	

### Clinical feature analysis

Univariate and multivariate Cox regression identified five independent clinical predictors of overall survival (all *p* < 0.05): liver metastasis (HR = 0.66, 95% CI: 0.456–0.955), TNM stage (HR = 60.58, 95% CI: 18.721–196.034), body weight (HR = 0.969, 95% CI: 0.948–0.989), number of HIFU sessions (HR = 0.852, 95% CI: 0.776–0.935), and treatment regimen (HR = 0.194, 95% CI: 0.194–0.331) (Table 2). These variables were used to construct the clinical prediction model and nomogram.

**Table 2:** Univariable and multivariable analysis of clinical features

Feature name	HR	lower 95%CI	upper 95%CI	*P*	HR	lower 95%CI	upper 95%CI	*p*-Value
Gender	1.060	0.791	1.420	0.697				
Age	0.997	0.974	1.021	0.809				
Location	1.078	0.952	1.222	0.236				
Echo	0.883	0.752	1.036	0.128				
Texture	0.992	0.647	1.521	0.97				
Boundary	0.927	0.692	1.244	0.615				
Shape	0.936	0.682	1.284	0.682				
Diameter	1.001	0.993	1.009	0.807				
Size	1.000	1.000	1.000	0.985				
Liver metastasis	0.689	0.517	0.919	< 0.05	0.66	0.456	0.955	< 0.05
Lymph node metastasis	0.963	0.720	1.288	0.797				
Vascular invasion	0.921	0.663	1.279	0.625				
TNM	15.748	9.284	26.711	< 0.05	60.58	18.721	196.034	< 0.05
KPS	0.987	0.972	1.003	0.114				
NRS	1.060	1.002	1.121	< 0.05	1.006	0.899	1.126	0.915
NRS score one month after treatment	1.091	1.018	1.170	< 0.05	0.976	0.842	1.132	0.749
CA199	1.000	1.000	1.000	< 0.05	1	1	1	0.969
CA125	1.000	1.000	1.000	0.746				
Weight	0.980	0.963	0.998	< 0.05	0.969	0.948	0.989	< 0.05
Height	0.421	0.070	2.520	0.344				
BMI	0.939	0.880	1.002	0.058				
HIFU cycle count	0.863	0.801	0.929	< 0.05	0.852	0.776	0.935	< 0.05
Treatment sequence	0.466	0.322	0.673	< 0.05	0.613	0.354	1.063	0.081
Jaundice	1.412	0.996	2.002	0.053				
Treatment regimen	0.290	0.238	0.354	< 0.05	0.253	0.194	0.331	< 0.05
Chemotherapy regimen	0.714	0.627	0.813	< 0.05	0.931	0.774	1.121	0.453

### Traditional radiomics feature extraction and model construction

A total of 1561 radiomics features were extracted from each ultrasound ROI (Additional Table 1). After Z-score normalization, features were screened using Pearson correlation (removing highly correlated pairs) and univariate Cox regression (*p* < 0.05). LASSO-Cox regression further reduced dimensionality, retaining 18 non-zero coefficient features (Additional Fig. S1) for multivariate Cox model construction.

### Deep Learning radiomics model construction and evaluation

**Fig. 2:**
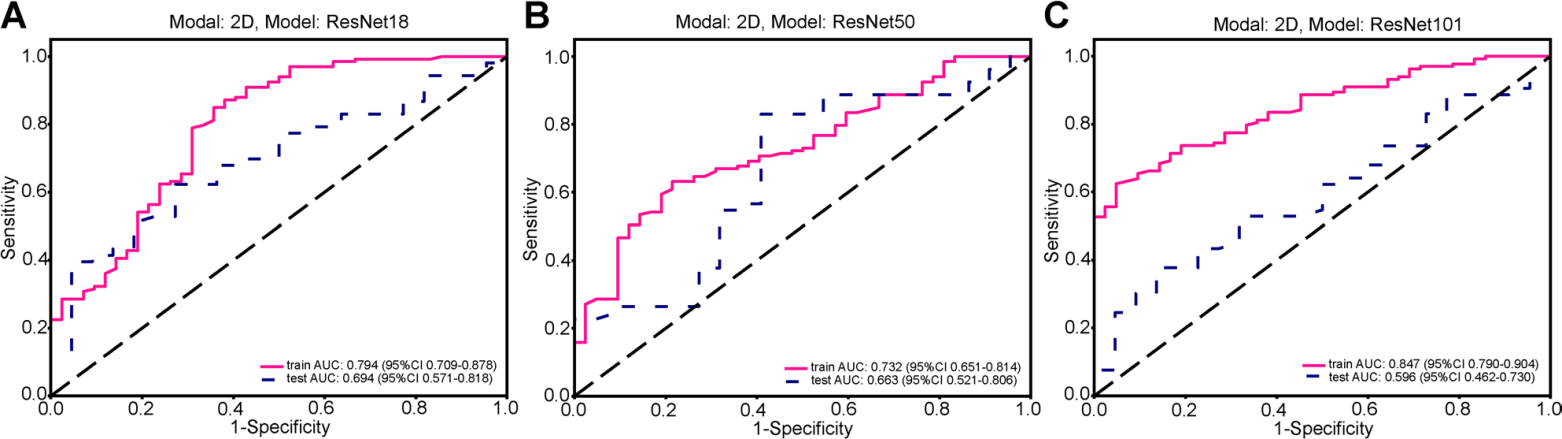
ROC results for deep learning signature of different models. **A** ROC curves of the deep learning signature based on the ResNet18 model in the training and validation cohorts. **B** ROC curves of the deep learning signature based on the ResNet50 model in the training and validation cohorts. **C** ROC curves of the deep learning signature based on the ResNet101 model in the training and validation cohorts

Transfer learning with CNN architectures (ResNet18/50/101) was used to build deep learning radiomics models. ResNet18 achieved the best performance in the training cohort (Accuracy 0.800, AUC 0.794, 95% CI: 0.709–0.878) and stable generalization in the test cohort (AUC 0.694, 95% CI: 0.571–0.818; specificity 0.955). ResNet50 and ResNet101 showed lower test AUCs (0.663 and 0.596, respectively). ResNet18 was selected as the backbone network for subsequent survival analysis feature extraction due to its balanced performance (Fig. 2; Table 3). Deep learning feature selection and visualization are shown in Additional Fig. S2.

**Table 3:** Metric results for deep learning radiomics signature

Model Name	Accuracy	AUC	95% CI	Sensitivity	Specificity	PPV	NPV	Cohort
ResNet18	0.800	0.794	0.7090–0.8780	0.850	0.643	0.883	0.574	train
ResNet18	0.560	0.694	0.5706–0.8179	0.396	0.955	0.955	0.396	test
ResNet50	0.669	0.732	0.6509–0.8138	0.632	0.786	0.903	0.402	train
ResNet50	0.760	0.663	0.5209–0.8058	0.830	0.591	0.830	0.591	test
ResNet101	0.703	0.847	0.7900-0.9043	0.624	0.952	0.976	0.444	train
ResNet101	0.520	0.596	0.4619–0.7302	0.377	0.864	0.870	0.365	test

#### Model interpretability analysis

**Fig. 3:**
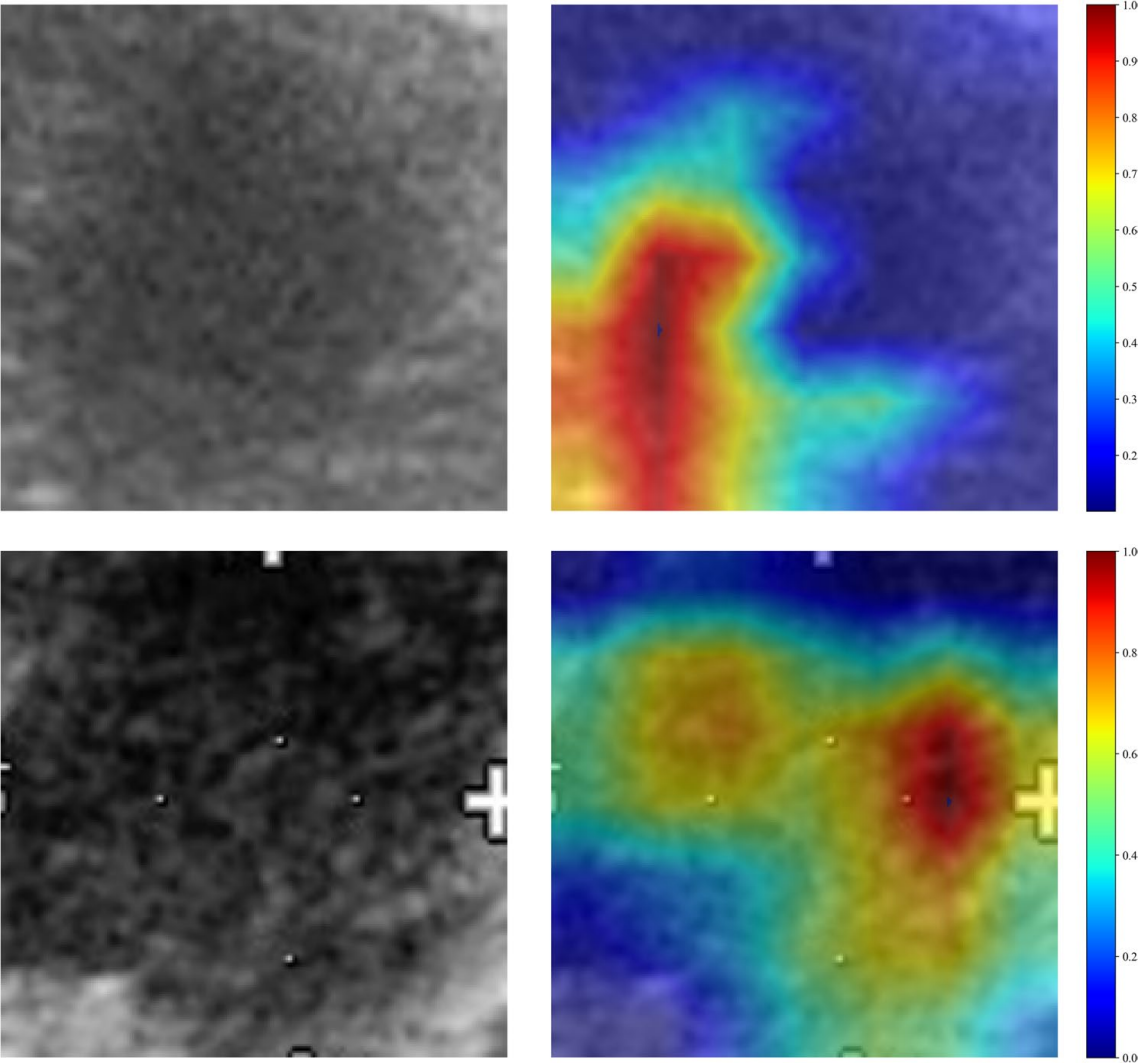
Presents the Grad-CAM visualizations for two typical samples. These visualizations are instrumental in demonstrating how the model focuses on different regions of the images for making its predictions

Gradient-weighted Class Activation Mapping (Grad-CAM) was used to visualize the trained deep learning model. Saliency maps were generated from gradient information of the final convolutional layer. As shown in Fig. 3, Grad-CAM identified key ultrasound regions most relevant to prognosis prediction, closely associated with tumor biology and treatment response. This visualization enhances model interpretability and clinical credibility.

### Model survival prediction performance evaluation

**Fig. 4:**
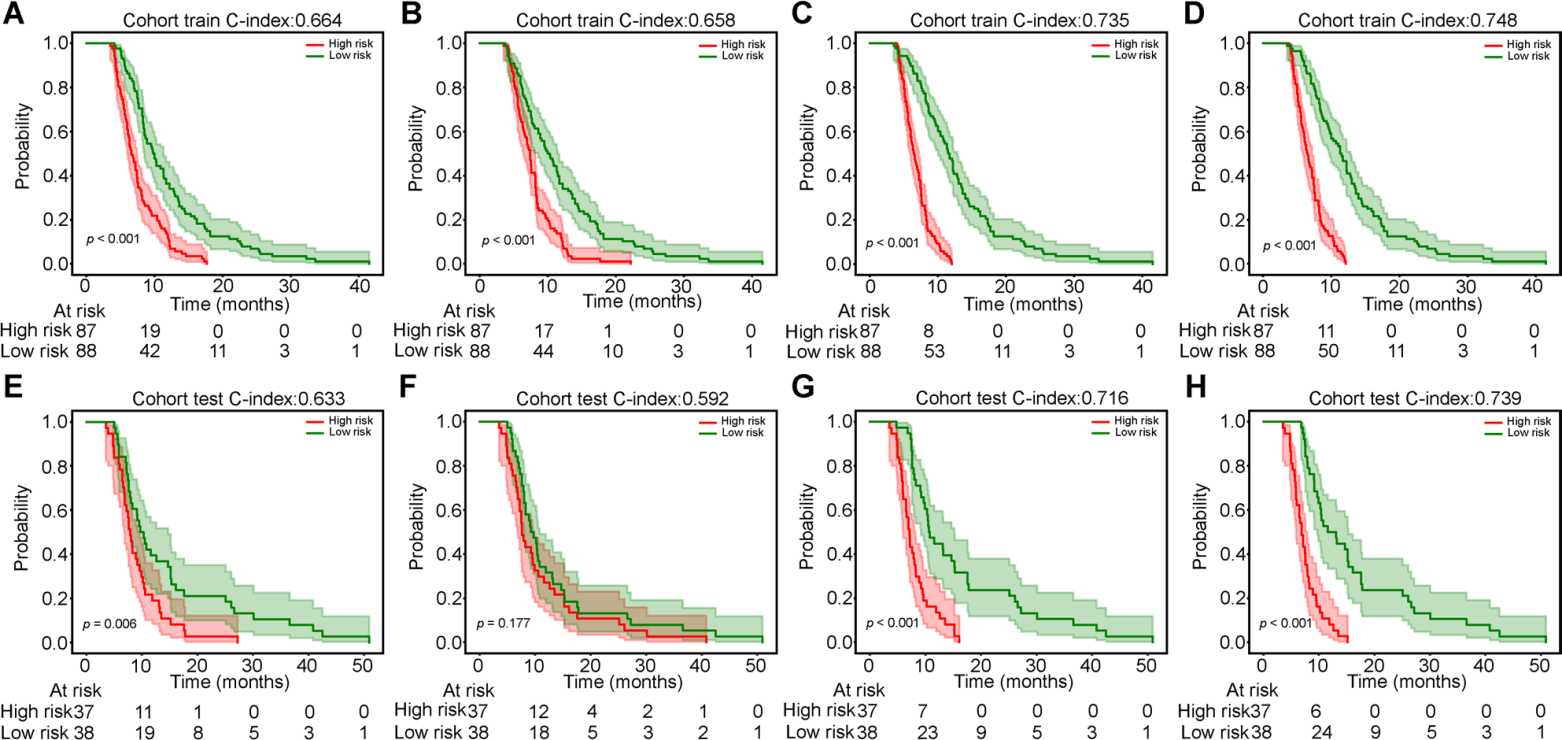
Kaplan-Meier curves for overall survival based on different models. Panels **A**-**D** show results for the training cohort: **A** Clinical model, **B** Radiomics model, **C** DLRadiomics model, and **D** Combined model. Panels **E**-**H** show results for the validation cohort: **E** Clinical model, **F** Radiomics model, **G** DLRadiomics model, and **H** Combined model

In the training cohort, the Combined Model showed the highest C-index (0.748, 95% CI: 0.684–0.813), followed by Deep Learning Radiomics (0.735), Clinical (0.664), and Traditional Radiomics (0.658) (Table 4; Fig. 4). A consistent trend was observed in the test cohort: Combined Model (0.739, 95% CI: 0.639–0.838), Deep Learning Radiomics (0.716), Clinical (0.633), and Traditional Radiomics (0.592). Deep learning radiomics features demonstrated superior prognostic value, and integration with clinical features further improved model generalization and robustness.

**Table 4:** C-index of different signatures

Clinical	Radiomics	DLRadiomics	Combined	Cohort
0.664 (0.594–0.734)	0.658 (0.587–0.728)	0.735 (0.669-0.800)	0.748 (0.684–0.813)	train
0.633 (0.523–0.742)	0.592 (0.481–0.703)	0.716 (0.614–0.818)	0.739 (0.639–0.838)	test

### Time-dependent ROC analysis

**Fig. 5:**
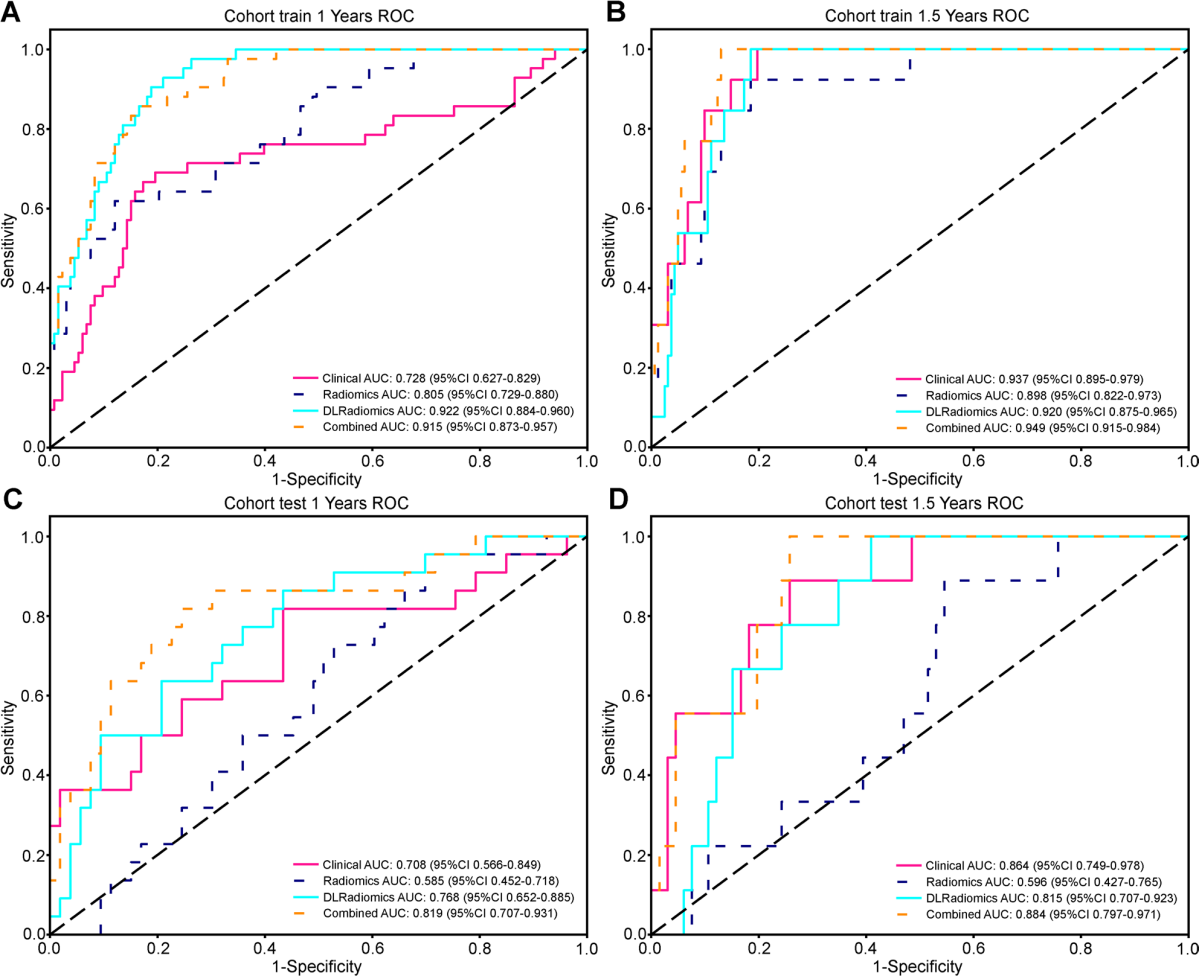
Time-dependent ROC curves for predicting survival outcomes in the training and validation cohorts. The models' predictive performance was assessed at different time points. **A** ROC curves for predicting 1-year survival in the training cohort, and **B** in the validation cohort. **C** ROC curves for predicting 1.5-year survival in the training cohort, and **D** in the validation cohort

**Fig. 6:**
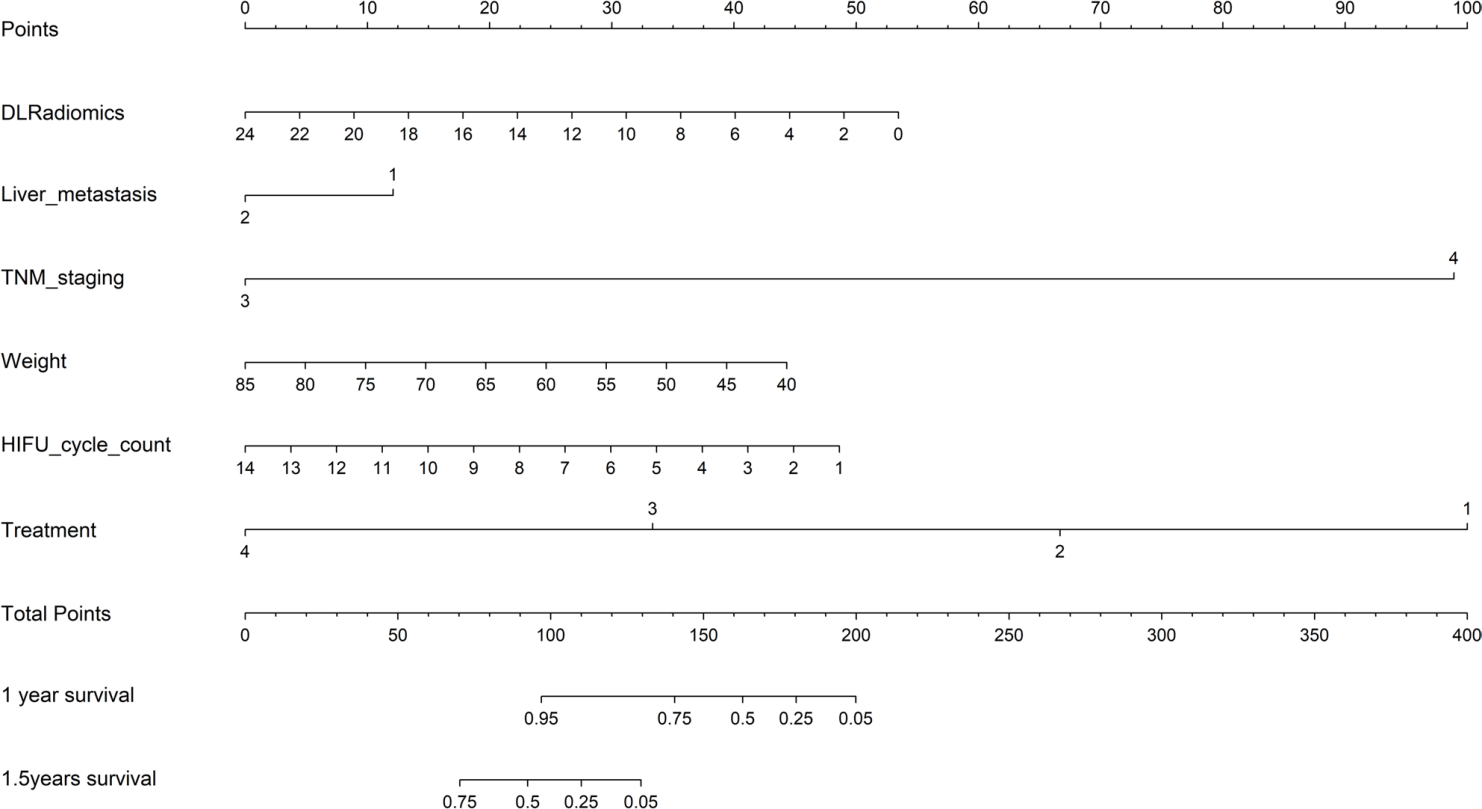
A radiomics-based nomogram integrating deep learning signatures and clinical factors for predicting prognosis in patients with advanced pancreatic cancer

Time-dependent ROC analysis evaluated model performance for 1- and 1.5-year survival (Fig. 5; Table 5). For 1-year: in training, DLRadiomics had the highest AUC (0.922), sensitivity 0.929, NPV 0.972; Combined Model AUC 0.915; Radiomics 0.805; Clinical 0.728. In test, Combined Model led (AUC 0.819), followed by DLRadiomics (0.768); Radiomics 0.585; Clinical 0.708. For 1.5-year: in training, Combined Model performed best (AUC 0.949), then DLRadiomics (0.920); Radiomics and Clinical lower. In test, Combined Model maintained superiority (AUC 0.884), followed by DLRadiomics (0.815) and Clinical (0.864); Radiomics 0.596. The Combined Model showed the most stable discrimination. A nomogram integrating deep learning radiomics and clinical predictors (body weight, liver metastasis, TNM stage, HIFU sessions, treatment regimen) was constructed for prognosis assessment (Fig. 6).

**Table 5:** Metrics in train and test cohorts for predicting the risk of survival by different models

Signature	Accuracy	AUC	95% CI	Sensitivity	Specificity	PPV	NPV	Survival	Cohort
Clinical	0.777	0.728	0.6272–0.8290	0.690	0.805	0.527	0.892	1 Years	Train
Radiomics	0.817	0.805	0.7290–0.8804	0.619	0.880	0.619	0.880	1 Years	Train
DLRadiomics	0.823	0.922	0.8840–0.9603	0.929	0.789	0.582	0.972	1 Years	Train
Combined	0.846	0.915	0.8729–0.9574	0.857	0.842	0.632	0.949	1 Years	Train
Clinical	0.640	0.708	0.5662–0.8489	0.818	0.566	0.439	0.882	1 Years	Test
Radiomics	0.480	0.585	0.4516–0.7182	0.955	0.283	0.356	0.937	1 Years	Test
DLRadiomics	0.653	0.768	0.6521–0.8848	0.864	0.566	0.452	0.909	1 Years	Test
Combined	0.773	0.819	0.7069–0.9312	0.818	0.755	0.581	0.909	1 Years	Test
Clinical	0.817	0.937	0.8947–0.9790	1.000	0.802	0.289	1.000	1.5 Years	Train
Radiomics	0.823	0.898	0.8224–0.9734	0.923	0.815	0.286	0.992	1.5 Years	Train
DLRadiomics	0.829	0.920	0.8750–0.9655	1.000	0.815	0.302	1.000	1.5 Years	Train
Combined	0.880	0.949	0.9148–0.9836	1.000	0.870	0.382	1.000	1.5 Years	Train
Clinical	0.760	0.864	0.7490–0.9783	0.889	0.742	0.320	0.980	1.5 Years	Test
Radiomics	0.507	0.596	0.4266–0.7653	0.889	0.455	0.182	0.968	1.5 Years	Test
DLRadiomics	0.640	0.815	0.7068–0.9228	1.000	0.591	0.250	1.000	1.5 Years	Test
Combined	0.773	0.884	0.7967–0.9709	1.000	0.742	0.346	1.000	1.5 Years	Test

## Discussion

Managing elderly patients with advanced pancreatic cancer remains challenging due to comorbidities, reduced organ reserve, and poor chemotherapy tolerance. HIFU, a non-invasive, repeatable technique recognized by the NIH in 2013 for unresectable pancreatic cancer [42], requires no general anesthesia, enables quick recovery, and is suitable for elderly or frail patients. HIFU disrupts tumor stroma, enhances chemotherapy penetration [42, 43]. alleviates pain, and synergizes with systemic therapy. HIFU plus chemotherapy improves efficacy over chemotherapy alone [44, 45]. Iodine-125 seed implantation is a conformal, minimally invasive internal radiotherapy ideal for elderly patients unable to tolerate fractionated radiotherapy. HIFU combined with chemotherapy or radiotherapy yields superior clinical benefit and survival versus HIFU alone [45, 46].

Supportive and integrated care models are crucial for elderly patients. Tumor heterogeneity drives variable treatment responses, necessitating effective, low-toxicity strategies and individualized selection. This study used machine learning and deep learning to construct a combined model integrating deep learning radiomics and clinical features to predict prognosis in elderly advanced pancreatic cancer patients receiving HIFU-based treatment.

Reliable prognostic biomarkers beyond CA19-9 are lacking [47, 48]. Ultrasonography is convenient and non-invasive [49], but traditional features insufficiently capture tumor biology. Radiomics characterizes tumor heterogeneity and aids prognosis [50–52]. Models combining radiomics and clinical parameters perform well in various cancers [53, 54], but ultrasound radiomics in pancreatic cancer prognosis remains scarce. Shao et al. [55] and Zhang et al. [56] both developed radiomics nomograms based on MRI to predict prognosis following HIFU therapy for pancreatic cancer. Although this imaging modality offers excellent image quality, approximately 15–20% of elderly patients have contraindications to gadolinium-based contrast agents due to renal insufficiency. Moreover, MRI is associated with high costs, long appointment waiting times, and limited accessibility. In contrast, ultrasound offers advantages including no radiation, no need for contrast agents, bedside operability, and low cost, making it a more acceptable imaging modality for elderly patients with pancreatic cancer. However, current applications of ultrasound radiomics in pancreatic cancer remain predominantly focused on differential diagnosis between benign and malignant lesions [53], with a notable paucity of studies on prognostic prediction following HIFU therapy. The present study introduces ultrasound-based deep learning radiomics into this task, providing a feasible alternative prognostic assessment tool for elderly patients who are intolerant or unsuitable for MRI. Furthermore, both prior studies [55, 56] employed conventional handcrafted radiomics paradigms, relying on predefined feature libraries and LASSO-based feature selection, which are subject to inherent limitations such as restricted feature representation capacity and high risk of overfitting. In contrast, the present study constructed a deep learning radiomics model using ResNet18 as the backbone architecture, enabling end-to-end learning of high-dimensional prognostic features through automated feature extraction. Our results demonstrated that the DLRadiomics model achieved a significantly higher C-index (0.716) in the test cohort compared to the conventional handcrafted radiomics model (0.658), which is highly consistent with the findings of Guo et al. [57] in soft tissue sarcoma (CNN-derived features outperforming handcrafted features). This study provides empirical evidence supporting a methodological paradigm shift from handcrafted feature engineering to deep learning-based automated feature extraction in the field of prognostic prediction for pancreatic cancer patients treated with HIFU.

Deep learning excels in medical image analysis. “Radiomics + deep learning” enables automatic feature extraction and improves predictive reliability [58]. CNNs outperform traditional methods in lesion detection, segmentation, and prognosis. CNN-based PET/CT radiomics predicted advanced nasopharyngeal carcinoma prognosis with high performance [59]; Guo et al. [57] integrated clinical parameters with CNN-derived MRI features for soft tissue sarcoma grading, outperforming single models (AUC 0.834 vs. 0.662). In this study, DLRadiomics (ResNet18) achieved a test C-index of 0.716; the combined model integrating clinical features reached 0.739. At 1 and 1.5 years, the combined model maintained the highest AUCs (0.819 and 0.884). DLRadiomics features offer superior prognostic value; combining with clinical features optimizes performance and stability. The combined model and nomogram enhance quantification of tumor heterogeneity, improve sensitivity–specificity balance, and aid high-risk patient identification.

Although deep learning features lack direct physical or biological definitions, when combined with Grad-CAM heatmap visualizations, we observed that the model’s high-activation regions often corresponded to irregular tumor margins, internal hypoechoic areas, and indistinct interfaces between the tumor and surrounding tissue. In clinical practice, these imaging features are frequently associated with aggressive tumor growth, desmoplastic stroma, necrosis, or microvascular invasion. While this interpretation remains speculative, it suggests that the deep learning model may capture underlying imaging phenotypes reflective of the biological aggressiveness of the tumor.

Univariate and multivariate Cox regression identified body weight, liver metastasis, TNM stage, HIFU sessions, and treatment regimen (all *p* < 0.05) as independent prognostic factors. These vary across studies [56, 60–62], reflecting population and methodological heterogeneity, indicating context-specific model construction.

These five predictors have clinical and biological relevance. Liver metastasis and TNM stage reflect tumor burden [61, 63, 64]. Weight loss indicates poor nutritional status and increased mortality risk [65]. Sarcopenia, prevalent in 20–65% of pancreatic cancer patients and higher in elderly, is linked to poorer survival [66–68]. Underweight (BMI < 18.5) is a classic sarcopenia risk factor; nutritional support may improve prognosis.

HIFU session number and treatment regimen reflect treatment intensity. Adequate sessions improve local control and survival. HIFU is well-tolerated and repeatable [69]. When the treatment effect is stable disease or progression, the interval for repeated HIFU treatments at the same site is conventionally more than 4 weeks, typically repeated every 1–3 months [70]. Repeated sessions prolong survival without increasing adverse events [71–74]; Multi-modal therapy (HIFU + seeds, HIFU + chemotherapy) enhances efficacy. HIFU plus radiotherapy shows synergy [72–74]; our preliminary data showed median OS 13.0 months (HIFU+seed) vs. 9.5 months (HIFU alone, *p* < 0.001). HIFU plus chemotherapy enhances drug penetration [72]. our data showed median OS 12.4 months vs. 8.9 months (*p* = 0.008), consistent with prior studies [18, 20, 75]; HIFU-based combination therapy provides superior survival benefits. However, it should be noted that the intensity of the treatment regimen and the number of HIFU sessions may themselves be influenced by the length of patient survival, treatment tolerance, and symptom control. Patients who survive longer objectively have more opportunities to undergo additional HIFU sessions. Therefore, although these variables demonstrated prognostic discriminatory ability in the model, they should be interpreted with caution and should not be regarded as direct, causal markers of treatment benefit.

These clinical factors inform disease characteristics, physical status, and treatment strategy. Their inclusion enhances prognostic accuracy and underscores the importance of nutritional support, burden assessment, and individualized multi-modal therapy. The combined model outperformed single-modality models, indicating complementary value of deep learning radiomics and clinical factors. The nomogram enables intuitive risk stratification and treatment optimization. From a practical perspective, the model developed in this study is not intended to directly guide specific therapeutic decisions, but rather to serve as a pre-treatment risk stratification tool to assist clinical decision-making. For example, the model can be used prior to HIFU treatment to identify patients with extremely poor prognosis, providing a reference for physician-patient communication, treatment intensity selection, and early palliative care intervention. In the future, through embedded software or cloud-based platforms, this model could potentially be integrated with ultrasound devices to enable real-time, low-cost bedside risk assessment.

This study has limitations. It is a single-center retrospective study requiring prospective, multi-center validation. Second, ultrasound-based radiomic features are highly sensitive to operator technique, probe position, equipment model, and image acquisition settings. Although all images in this study were obtained from a single center using the same equipment—which ensures internal consistency—it also limits the generalizability of the model. Therefore, rigorous external validation in multi-center, multi-device, and multi-operator settings is essential before broader application. Furthermore, this study performed manual segmentation on the single largest tumor slice. Although this approach is pragmatic and feasible in a retrospective setting, pancreatic tumors are highly heterogeneous, and single-slice analysis may not fully capture the overall complexity of the tumor, particularly for lesions with irregular morphology or large volume. Future studies should consider incorporating three-dimensional volumetric segmentation or deep learning-driven automatic/semi-automatic segmentation methods to more comprehensively characterize tumor heterogeneity. Deep learning feature interpretability and biological significance require further exploration. Future prospective multi-center studies integrating multi-omics (e.g., genomics, transcriptomics) are needed to reveal underlying mechanisms and enhance interpretability, ultimately enabling imaging–molecular integration for precise stratification and individualized treatment. Predictive performance for long-term survival (> 1.5 years) remains uncertain due to follow-up duration, limiting very long-term clinical utility. Additionally, potential interactions and non-linear relationships between clinical factors and deep learning radiomics features may not be fully captured by the current modeling approach. Finally, the study population was restricted to elderly patients aged ≥ 65 years. Future research will aim to extend the current deep learning radiomics framework to a full-age-spectrum cohort and further validate the model’s generalizability and robustness across different age groups through cross-age subgroup analyses and propensity score matching.

## Conclusion

This study developed and evaluated a combined prediction model and nomogram tool integrating deep learning radiomics features with clinical risk factors. This model can extract high-dimensional information from ultrasound images, demonstrated favorable prognostic prediction performance, showing potential suitability for the clinical prognosis assessment of elderly patients with advanced pancreatic cancer undergoing HIFU treatment. This tool could aid in achieving precise risk stratification and individualized treatment decisions for elderly advanced pancreatic cancer patients, provides a potential evidence-based reference for formulating comprehensive pancreatic cancer treatment strategies, and may hold promise for contributing to improved survival prognosis of pancreatic cancer patients. The deep learning radiomics nomogram proposed in this study demonstrated favorable prognostic stratification ability in the internal validation cohort. However, this study remains at a retrospective, single-center stage. This model should be regarded as a potential prognostic assessment tool, and rigorous multi-center external validation as well as prospective cohort studies are required to confirm its stability and generalizability before clinical implementation.

## Supplementary Information

Supplementary Material 1. This is a docx file named "Additional Files", which primarily presents additional features, including Additional Fig. S1 Radiomic Feature Selection and Visualization Analysis and Additional Fig. S2 Deep Learning Feature Selection and Visualization Analysis

Supplementary Material 2. This is a CSV file named "Additional Table 1 rad_features_US.csv", which presents a total of 1561 radiomics features extracted from the ultrasound region of interest (ROI) of each patient

Supplementary Material 3

## Data Availability

The datasets presented in this article are not readily available because the data analyzed in this study is subject to the following glicenses/restrictions: The datasets for this article are not publicly available as it is private data that belongs to Huadong Hospital Affiliated to Fudan University. Requests to access the datasets should be directed to corresponding author. Requests to access the datasets should be directed to Hong Zhao, hongzhhdyy@163.com.

## Abbreviations

HIFU: High-intensity focused ultrasound
OS: Overall survival
KPS: Karnofsky performance status
NRS: Numerical rating scale
ROI: Region of interest
IBSI: Image Biomarker Standardisation Initiative
GLCM: Gray level co-occurrence matrix
GLRLM: Gray level run length matrix
GLSZM: Gray level size zone matrix
NGTDM: Neighboring gray tone difference matrix
LoG: Laplacian of Gaussian
LASSO: Least absolute shrinkage and selection operator
SGD: Stochastic gradient descent
HR: Hazard ratio
CNNs: Convolutional Neural Networks
AUC: Area under the curve
DCA: Decision curve analysis
C-index: Concordance index
Grad-CAM: Gradient-weighted Class Activation Mapping
ROC: Receiver operating characteristic
DLRadiomics: Deep learning radiomics
NIH: National Institutes of Health
