# Supplementary material for: Development and validation of a deep learning radiomics model based on ultrasound and clinical features to predict prognosis in elderly patients with advanced pancreatic cancer after HIFU therapy
Source: BMC Gastroenterol. 2026 Mar 5;26:223. doi: 10.1186/s12876-026-04716-6 (PMC13069712; doi:10.1186/s12876-026-04716-6)
Supplement: Supplementary file 1 — Supplementary Material 1. This is a docx file named "Additional Files", which primarily presents additional features, including Additional Fig. S1 Radiomic Feature Selection and Visualization Analysis and Additional Fig. S2 Deep Learning Feature Selection and Visualization Analysis [file 12876_2026_4716_MOESM1_ESM.docx]

### **Additional Files**

# **Manuscript Title:** Development and Validation of a Deep Learning Radiomics Model Based on Ultrasound and Clinical Features to Predict Prognosis in Elderly Patients with Advanced Pancreatic Cancer after HIFU Therapy

**Author Information：**Yumei Liu^1,3^, Yongshuo Ji^1^, Junqiu Zhu^1^, Linglin Zhu^1^, Yanfei Zhu^1^, Hong Zhao^1^*, Zhijun Bao^2-4^*

^1^High-Intensity Focused Ultrasound Center of Oncology Department, Huadong Hospital Affiliated to Fudan University, Shanghai, China; ^2^Department of Gerontology, Huadong Hospital Affiliated to Fudan University, Shanghai, China; ^3^Shanghai Key Laboratory of Clinical Geriatric Medicine, Shanghai, China; ^4^Shanghai Institute of Geriatrics and Gerontology, Shanghai, China

**Correspondence to*:

Professor Hong Zhao, High-Intensity Focused Ultrasound Center of Oncology Department, Huadong Hospital Affiliated to Fudan University, 139 West Yan’an Road, Jing’an, Shanghai 200000, China

Email: [hongzhhdyy@163.com](mailto:hongzhhdyy@163.com)

Professor Zhijun Bao, Department of Gerontology, Huadong Hospital Affiliated to Fudan University, 221 West Yan’an Road, Jing’an, Shanghai 200040, China

E-mail: ultramancomeon@163.com

# **1. Additional Data**

**Additional Table 1 rad_features_US.csv:** A total of 1561 radiomics features were extracted from the ultrasound ROI of each patient. Due to its large size, the table has been included as a separate file named "Additional Table 1 rad_features_US.csv" in the supplementary materials.

### ****2. Additional Features****


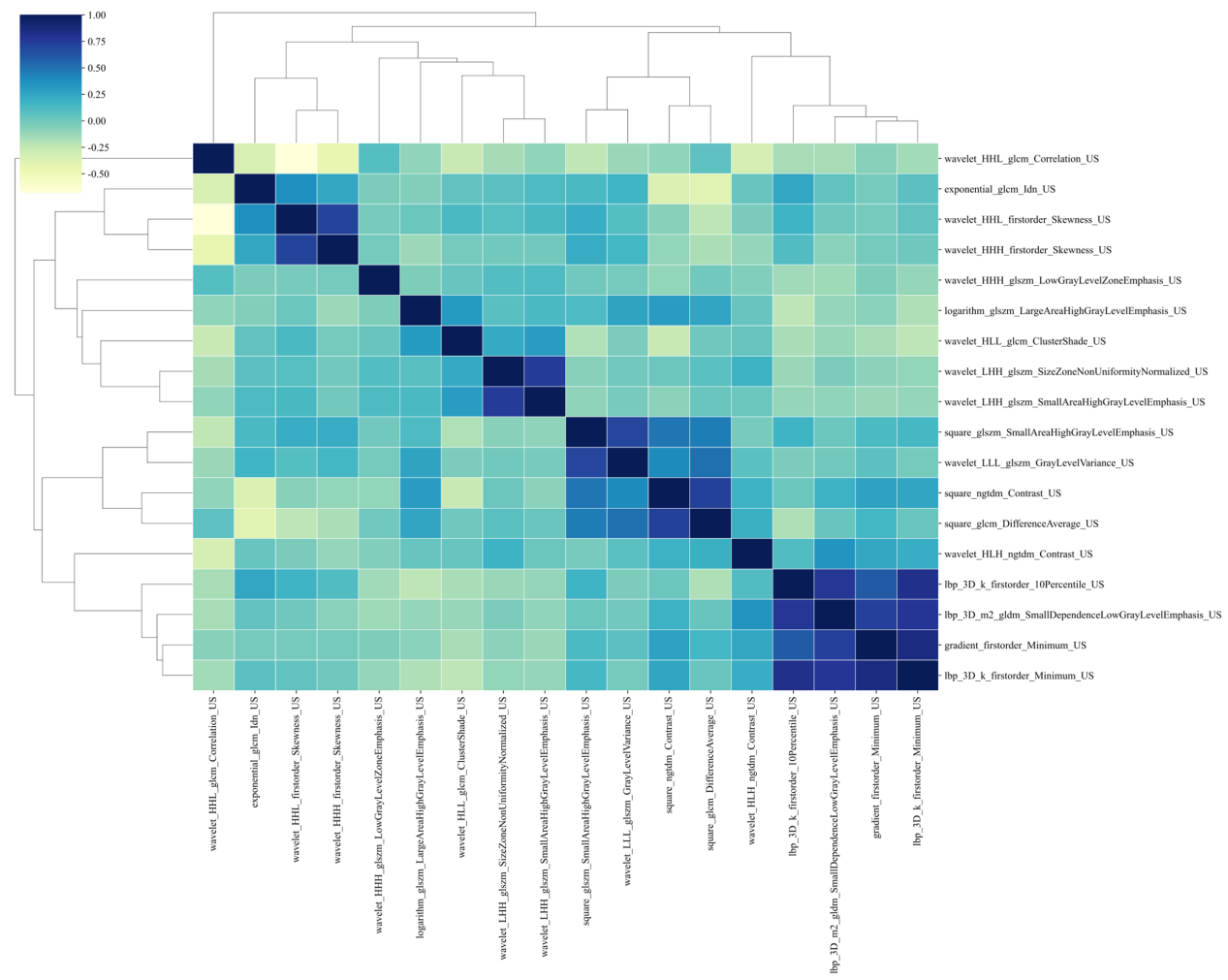


**A**


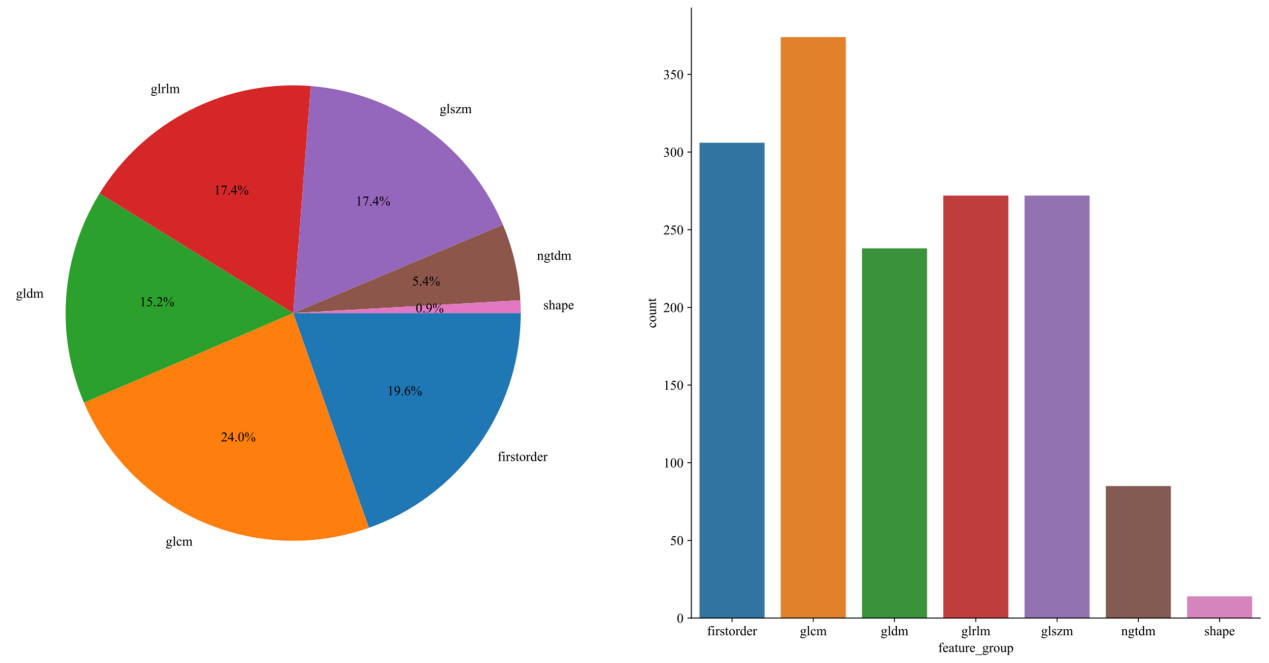


**B**


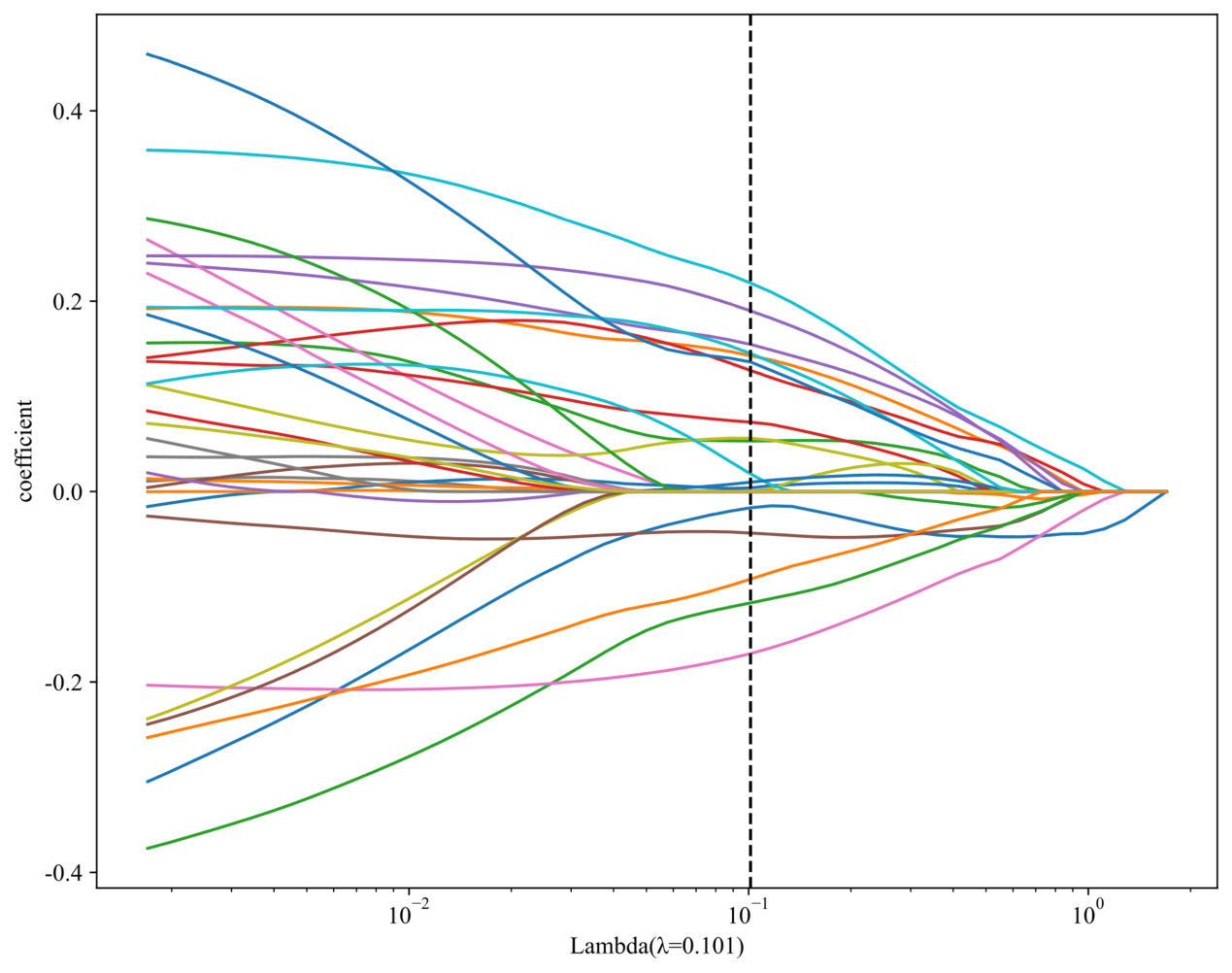


**C**


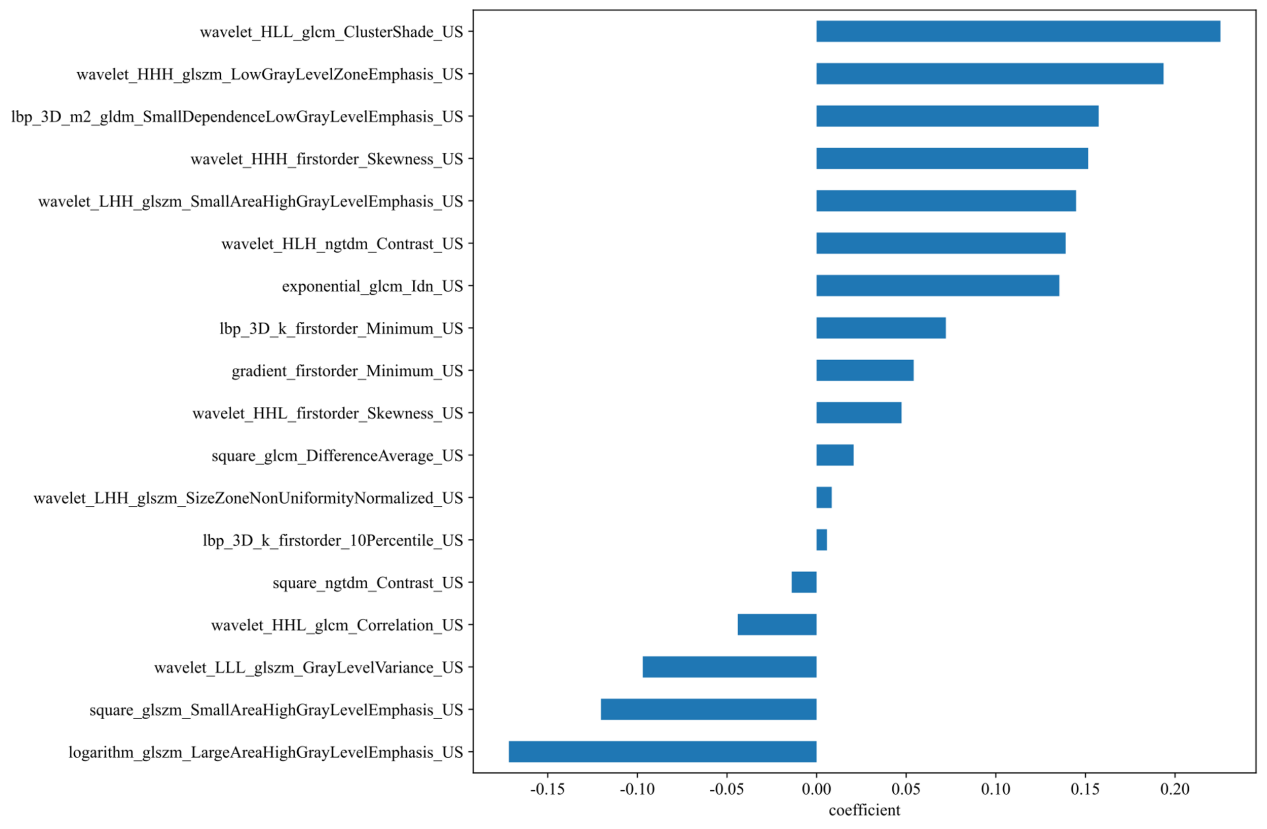


**D**

****Additional Fig. S1**** Radiomic Feature Selection and Visualization Analysis. Heatmap of the selected radiomic features (A), category distribution of handcrafted radiomic features (B), LASSO regression coefficient path diagram of radiomic features. Lines of different colors represent the coefficient changes corresponding to each feature (C), LASSO regression coefficients of the selected radiomic features after screening (D).


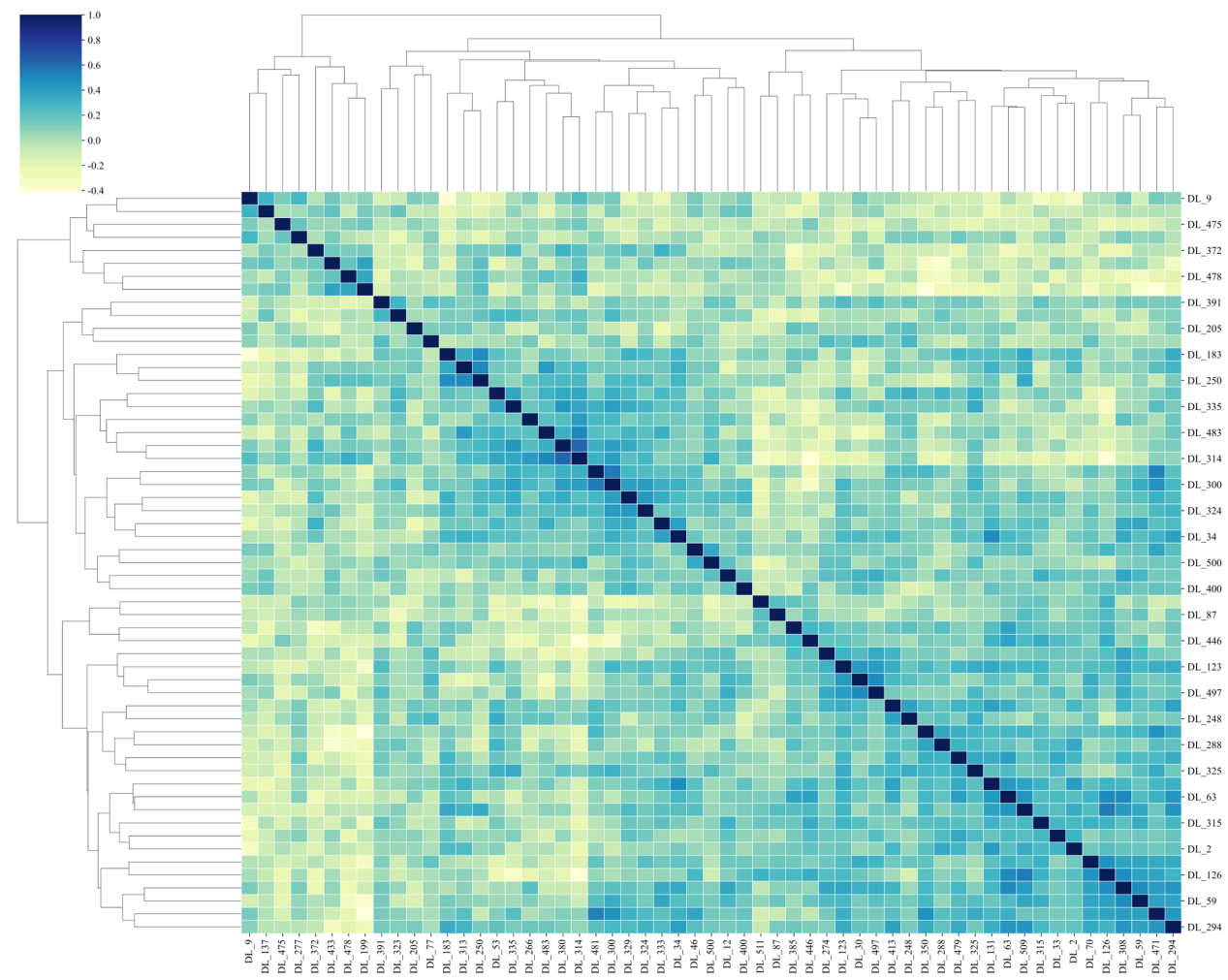


**A**


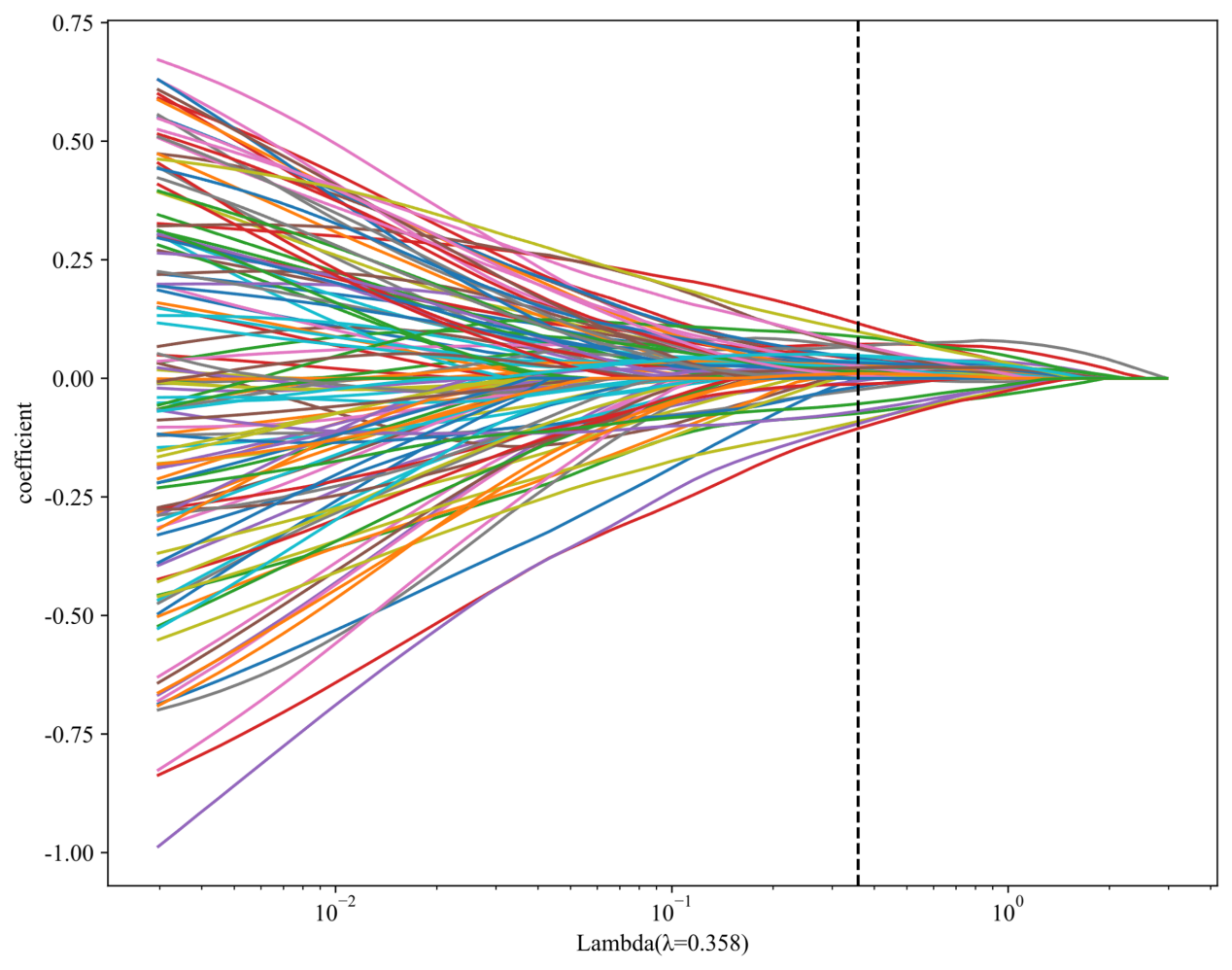


**B**


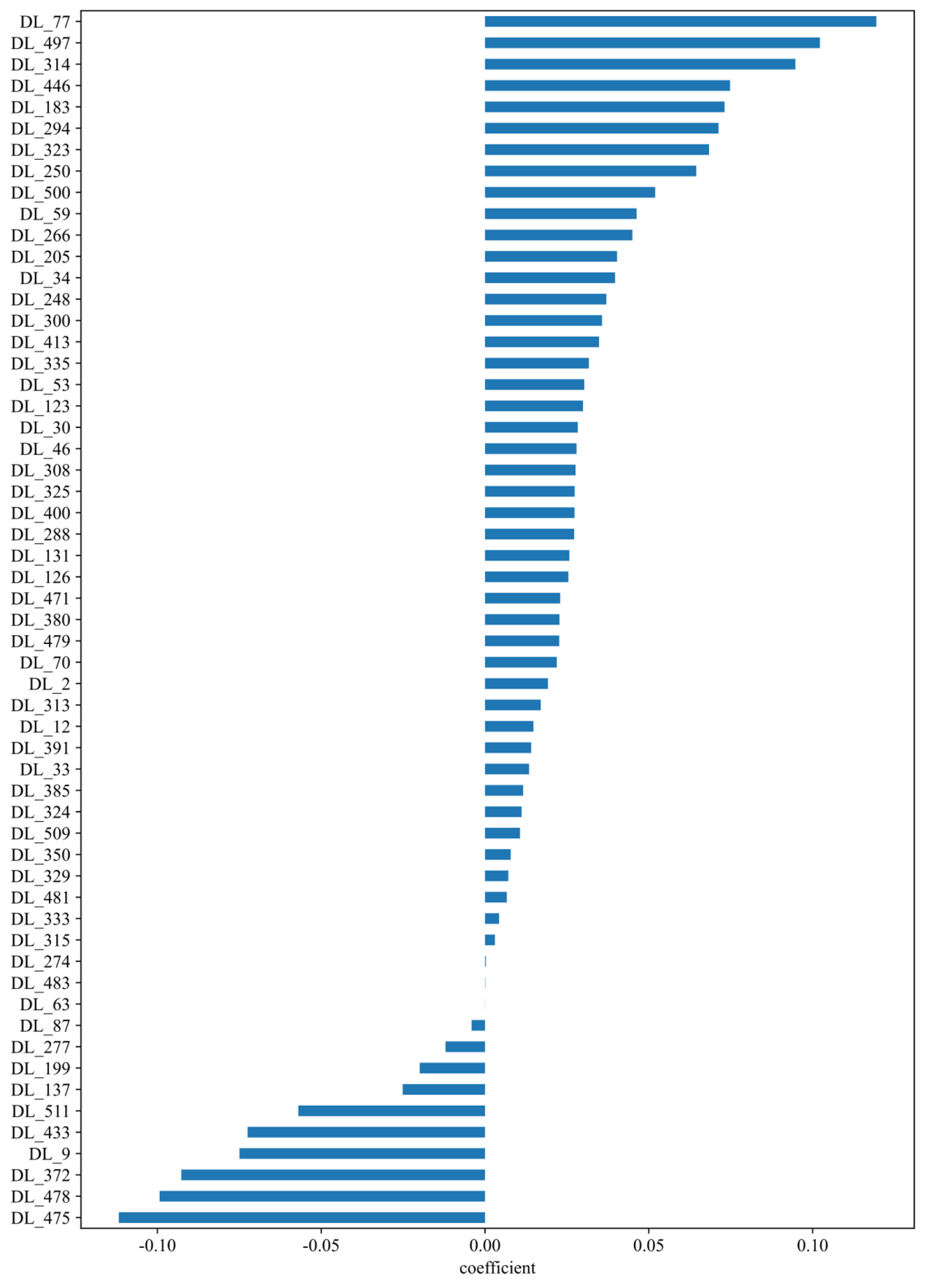


**C**

****Additional Fig. S2** Deep Learning Feature Selection and Visualization Analysis. Heatmap based on the selected deep learning radiomic features, showing the clustering analysis results of cases and features (A); LASSO regression coefficient path diagram of the deep learning radiomic features (B); LASSO regression coefficients of the selected deep learning radiomic features after screening (C).**
